# Constellation Plots in KNIME: An Automated Scaffold‐Based Workflow for Interactive Chemical Space Visualization

**DOI:** 10.1002/minf.70035

**Published:** 2026-04-30

**Authors:** Carlos D. Ramírez‐Márquez, Edgar López‐López, José L. Medina‐Franco

**Affiliations:** ^1^ DIFACQUIM Research Group Department of Pharmacy School of Chemistry Universidad Nacional Autónoma de México Mexico; ^2^ Department of Pharmaceutical Biosciences Uppsala University Uppsala Sweden

**Keywords:** Alzheimer's disease, chemical space, chemoinformatics, drug discovery, open science

## Abstract

Chemical space analysis is extensively used in different chemistry areas, ranging from the study of natural products to drug discovery projects. Its versatility stems from the ability to integrate continuous properties with molecular representations. This data is used to generate visualizations through dimensionality reduction algorithms. Constellation Plots have been proposed as a general approach to the visual representation of chemical space by encoding structural similarity, scaffold contents, frequency, and continuous properties into a single coordinate‐based map. Thus, Constellation Plots provide a high‐density visual representation of the chemical space of compound datasets with complex relations. Despite the versatility of Constellation Plots, there remains a significant lack of intuitive, user‐friendly, or low‐code protocols to automate the generation of these plots for non‐computational experts. Herein, we present an interactive and automated scaffold‐based Constellation Plot workflow developed within the open‐source platform KNIME, facilitating chemical space visualization and analysis. To illustrate the application of the workflow, we used a dataset of 5,211 compounds that inhibit Tau protein, a key therapeutic target for Alzheimer's disease. The KNIME workflow is a general resource that can be used to analyze virtually any data set annotated with a property, including biological activity. The workflow is freely available at: https://github.com/Daniphantom99/KNIME_Constellation_plots.

## Graphical abstract

The interactive, automated scaffold‐based Constellation Plot workflow provides a high‐density visual representation of the chemical space of compound datasets with complex relationships.

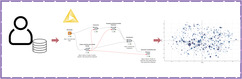

## 1 Introduction

The concept of chemical space is central to chemoinformatics [1] being the framework for numerous theoretical and methodological developments [2, 3, 4, 5]. Although chemical space analysis has been most extensively applied in drug discovery and the identification of bioactive compounds, its use extends to other areas of chemistry, including agrochemicals, natural products, food chemistry, and materials science [6]. A key strength of chemical space analysis lies in its visual representation, particularly given the high dimensionality of molecular descriptors and structural features. Two‐ and three‐dimensional projections of chemical space are widely used for diversity analysis of compound libraries, library design, compound selection, and the exploration of structure–activity relationships (SAR) and structure–property associations (SPA). The latter has been proposed as a broader conceptual extension of classical SAR analysis in drug discovery [7]. Because compound datasets are typically described using multiple descriptor types and representations, numerous approaches have been developed to generate meaningful low‐dimensional visualizations of complex chemical spaces [8, 9, 10].

Among these approaches, Constellation Plots have been proposed as a scaffold‐based strategy for the two‐dimensional visualization of compound datasets [11]. In these plots, compounds are grouped according to their scaffolds, which allows the study of their relationships and associations explored by molecular similarity calculations from their molecular fingerprints, enabling the simultaneous visualization of structural properties within a single coordinate‐based map. Constellation Plots have been successfully applied to the exploration of SAR in kinase inhibitors [11], tubulin inhibitors [12], and, more recently, to the analysis of the chemical diversity of natural products [13].

Compared to traditional visualization methods such as Principal Component Analysis (PCA) or other coordinate‐based maps, Constellation Plots offer a distinct advantage by providing a structural‐hierarchical organization. By clustering molecules based on Bemis‐Murcko scaffolds [14], this approach aligns with the ‘SAR‐driven’ logic of medicinal chemistry, enabling immediate identification of scaffold diversity and frequency while reducing the visual noise often found in standard scatter plots. However, notable limitations include their sensitivity to dimensionality reduction hyperparameters (e.g., t‐SNE perplexity), the inherent exclusion of acyclic compounds that lack a defined scaffold and metal containing compounds, which may require complementary analysis for a comprehensive view of the chemical space. However, despite their versatility, the practical implementation of Constellation Plots remains technically demanding and lacks an automated, user‐friendly framework accessible to non‐computational researchers.

To address this limitation, the main objective of this study is to develop and provide an automated, scaffold‐based Constellation Plot workflow implemented in the open‐source KNIME [15] platform that enables interactive and reproducible chemical space visualization. The workflow is designed to facilitate the adoption of Constellation Plots by both computational and non‐computational experts. As a demonstration of its utility, we apply the workflow to a dataset of Tau protein inhibitors annotated with experimental activity. Tau is a promising therapeutic target for the treatment of Alzheimer's disease since its abnormal aggregates (neurofibrillary tangles) are closely correlated to clinical symptoms [16, 17].

## 2 Materials and Methods

The workflow was implemented using KNIME Analytics Platform (v. 5.4.2), which can be downloaded for free at https://www.knime.com/downloads. The workflow integrates core KNIME Base Nodes alongside specialized open‐source extensions, including KNIME Statistics Nodes (Labs) [18], RDKit Nodes Feature [19], and KNIME SVG Support [20]. Specific details of the workflow use are available at https://github.com/Daniphantom99/KNIME_Constellation_plots/blob/main/README.md.

### 2.1 Workflow General Description

**FIGURE 1:**
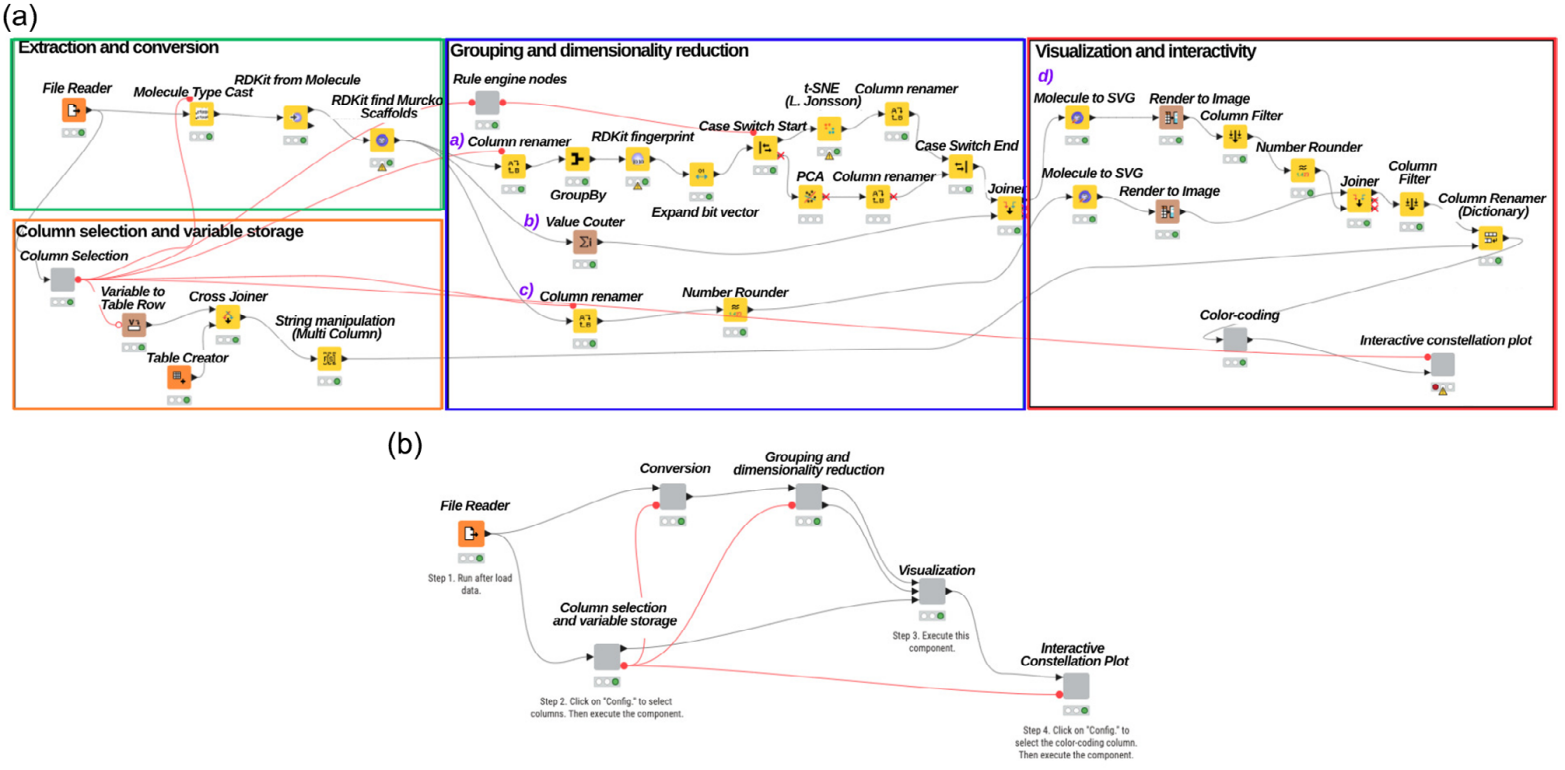
(a) Overview of the high‐level architecture used to generate Constellation Plots with KNIME. Modules are color‐coded to highlight core functional activities: *Extraction and Conversion* (green), *Column selection and variable storage* (orange), *Grouping and Dimensionality Reduction* (blue), and *Visualization and Interactivity* (red). Violet labels (a–d) designate specific workflow branches to facilitate systematic interpretation. (Technical details regarding node warnings and the handling of missing values are provided in the Note 1 ‐ Supporting Information). (b) End‐user workflow interface. The complete architecture is encapsulated into a single node and five specialized components for enhanced usability.

The workflow contains 33 distinct nodes and four specialized components with internal sub‐nodes. For illustrative purposes, the high‐level architecture is conceptualized into four modules based on their specific data‐processing tasks (Figure 1a). This modular design enabled the encapsulation of the entire pipeline into a single node and five high‐level functional components, resulting in a streamlined and user‐friendly interface for the end‐user (Figure 1b).

1. Extraction and Conversion (*green* area in Figure 1): In this initial stage, chemical information is retrieved from the source document. The linear chemical representations (e.g., SMILES [21] strings) are converted from string format into specific chemical objects, which are subsequently used to generate the *Bemis‐Murcko* scaffolds for each compound.
2. User column selection and variable storage (*orange*): This suite of nodes enables the user to select the specific data columns that will be processed throughout the workflow. Additionally, it allows for the selection of the dimensionality reduction method (PCA or t‐SNE). These parameters are internally captured and propagated as flow variables to ensure downstream consistency and algorithmic alignment.
3. Grouping and Dimensionality Reduction (*blue*): Scaffolds and physicochemical or biological properties are aggregated. Specific molecular fingerprints are calculated for each scaffold, followed by a dimensionality reduction technique (e.g., t‐SNE [22] (t‐distributed Stochastic Neighbor Embedding)), which will be used to visualize the chemical space. Simultaneously, a scaffold count is performed to maintain a reference of the non‐grouped dataset.
4. Visualization and Interactivity (*red*): Images are rendered from both the scaffolds and the original linear representations. Color‐coding based on specific numerical properties (e.g., potency or SlogP) is chosen by the user. Finally, an interactive component enables dynamic exploration, allowing users to synchronize data selection with the Constellation Plot generated.

The workflow is optimized for compound datasets of small organic compounds typically used in medicinal chemistry projects. In this case study of 5,211 compounds, execution is completed within minutes on standard workstations (e.g., 16 GB RAM). While the workflow remains efficient for larger datasets, users processing >50,000 compounds may experience increased RAM consumption during fingerprint generation and non‐linear increases in t‐SNE computation time. In such cases, adjusting KNIME's memory policy or hardware upgrades (32 GB RAM) are recommended to maintain interactivity.

### 2.2 Chemical Database Requirements

The current workflow described in this manuscript requires the user to provide a chemical database in any of the following formats: CSV, Table, XLSX, or SDF. The input file must be curated and include three columns: (1) a column with the compound's chemical linear representation (such as SMILES, SMARTS, or InChIKey); (2) a column with a numerical experimental or calculated property (for instance, a biological property such as activity or a physicochemical or molecular property), and (3) a column with the molecule name or an identifier. Of note, the column associated with a numerical property could include calculated scores if the Constellation Plot is used to analyze the results of a virtual screening.

To assist users in database curation, we provide an accessible Python‐based pipeline designed for Google Colab. This tool represents the direct translation of our research group's curation protocol into an interactive notebook format, available at: https://github.com/Daniphantom99/KNIME_Constellation_plots/blob/main/5_3_data_base_curation.ipynb.

### 2.3 Tau Inhibitors Dataset

As a case study to illustrate the application of the KNIME workflow to generate and analyze Constellation Plots, we used a public data set of small molecules, inhibitors of the Tau protein [23]. Tau´s pathological hyperphosphorylation and subsequent aggregation into neurofibrillary tangles are key hallmarks in Alzheimer's disease and related tautopathies. Consequently, a wide array of Tau aggregation inhibitors has been explored (e.g., CLR01 [24], Anle138b [25]), aiming to mitigate Tau‐related neurotoxicity. However, to date, no disease‐modifying drugs targeting these early aggregation stages have gained regulatory approval.

The data set is referred to as *Tau‐DS* to streamline its description throughout this manuscript. The Tau‐DS was retrieved from the ChEMBL database [26] (release ChEMBL_36) by filtering for the specific UniProt code: P10636 (microtubule‐associated protein Tau). Selection was based on reported potency values (nM) and the inherent ’Active’ metadata labels provided by the ChEMBL database for the confirmatory assays:

• qHTS for Inhibitors of Tau Fibril Formation, Fluorescence Polarization (Class of assay: confirmatory) [Related PubChem assays: 596]
• qHTS for Inhibitors of Tau Fibril Formation, Thioflavin T Binding (Class of assay: confirmatory) [Related PubChem assays: 596]

Duplicate entries were removed from the dataset, retaining only the instances with the lowest activity values to prioritize the most potent compounds. The dataset was curated following the workflow developed by Goméz‐García A. (2024) [27]. Tau‐DS contained 5,211 compounds, with potency values converted to μM.

## 3 Workflow

### 3.1 Interactive Scaffold‐Based Constellation Plot: Tau Inhibitors as a Case Study

The workflow (Figure 1a) begins with the *File Reader* node, from which users can select their chemical database from their computer in a particular format (CSV, SDF, or XLSX). Next, the ‘Column Selection’ component enables the user to specify the columns for SMILES strings, numerical property, and compound identifiers. These selections are converted into flow variables and passed to a transformation block consisting of *Variable to Table Row*, *Table Creator*, and *Cross Joiner* nodes. This setup dynamically generates a mapping table that is simultaneously parameterized into specific downstream analytical and visualization nodes, ensuring a standardized data schema throughout the entire workflow. Subsequently, the *Molecule Type Cast* and *RDKit From Molecule* nodes convert the chemical linear representation from string type to various molecular formats, such as Mol2, PDB, SDF, or, in this case, SMILES. The *RDKIT Find Murcko Scaffolds* uses the source column (SMILES column) to generate the scaffolds and append them into a new column (the workflow is configured to name this column as “Scaffold”). The first four nodes (Figure 1a ‐ *green*) are basically part of the *Workflow to identify Bemis‐Murcko Scaffolds* described in our previous study [28].

As a case study to generate a Constellation Plot, we used Tau‐DS, a chemical dataset containing compounds classified as “actives” against microtubule‐associated protein.

Branches description (Figure 1a ‐ Violet labels (a–d))

a. The *Column Renamer* node standardizes the user‐selected ’numerical property’ to ensure architectural consistency across the entire workflow. The *GroupBy* node groups the dataset based on the “Scaffold” column, and then takes the numerical property column (selected by the user in ’Column selection’) to perform different aggregation methods (Mean, Standard deviation, and Minimum were predefined). For this case of study the column “Activity μM” was selected from Tau‐DS. The output table includes one row for each combined value and a new column for each of the selected aggregation methods (e.g., “Property (Mean)”) (See Figure S1). Next, the RDKIT Fingerprint allows the fingerprint selection between Morgan, RDKit, MACCS, among others. For Tau‐DS, the Morgan fingerprints were selected, and the bit vector splitted to integer columns with the *Expand Bit Vector* node. The *Rule Engine nodes* component translates the user's selection into a control signal, facilitating the execution of the *CASE Switch Start* node. Consequently, depending on the chosen method, the workflow dynamically activates either the *t‐SNE* (*L. Jonsson*) [29] or the PCA node. Both nodes were configured to include from bitvector0 to bitvector1023 to reduce the dimensionality to 2 dimensions. Finally, the *Column Renamer* node standardizes the columns resulting from either node to ensure architectural consistency across the entire workflow (e.g., “t‐SNE dimension 0” to “Coord_X”).
b. The *Value Counter* node was used to count the number of occurrences of all scaffolds in the “Scaffold” column; the result column is named “count”. The branches “(a)” and “(b)” were then joined by the “Scaffold” and the “RowID” columns (the latest column name is assigned by the *Value counter* node, but contains scaffold information, therefore the join is possible). This new branch is “(d)”.
c. Last branch's objective is to change the original column name “Molecule CHEMBL ID” to “Original Structure” (*Column Renamer* node) for their usage in future nodes, and finally the *Number Rounder* node can be added if necessary (The “Activity (μM)” column from Tau‐DS was rounded to two decimal places).

Both branches “(c)” and “(d)” follow similar pathways: they are connected to an *RDKIT Molecule to SVG* node, which was preconfigured to:

• Select the column that contains RDKit Molecule object ‐ the “Scaffold” column for branch “d)”, “SMILES” column for the bottom one “(c)” ‐, and to
• Store the render in a new predefined internal column.

The *Render to Image* node was preconfigured to:

• Select the source column (that contains the render).
• Append the render (Image type: *png*) in a new predefined internal column.

The branch “(d)” is connected to a *Number Rounder* node (used for the same purpose described on “(c)”.

The *Joiner* node joins all the results into one table by the column “Scaffold” that both previous branches contained (including in the output the matching rows). Here, it is very important to assign the “(c)” branch as the “left table” and the “(d)” branch as the “right table”, since we need the original compound information to have a very high information interactive plot. The *Column Renamer* (*Dictionary*) node is linked to the *Column Filter* node, where the following columns are selected: ‘Original structure’, ‘Property (Mean)’, ‘Property (Standard deviation)’, ‘Property (Min*)’, ‘Coord_X’, ‘Coord_Y’, ‘count’, and ‘Scaffold Structure’; it is also connected to a set of transformation nodes (Figure 1 ‐ orange). This configuration restores the user's original numerical experimental or calculated property name, ensuring that no metadata is lost; thus, the final output remains consistent with the original input file.

**FIGURE 2:**
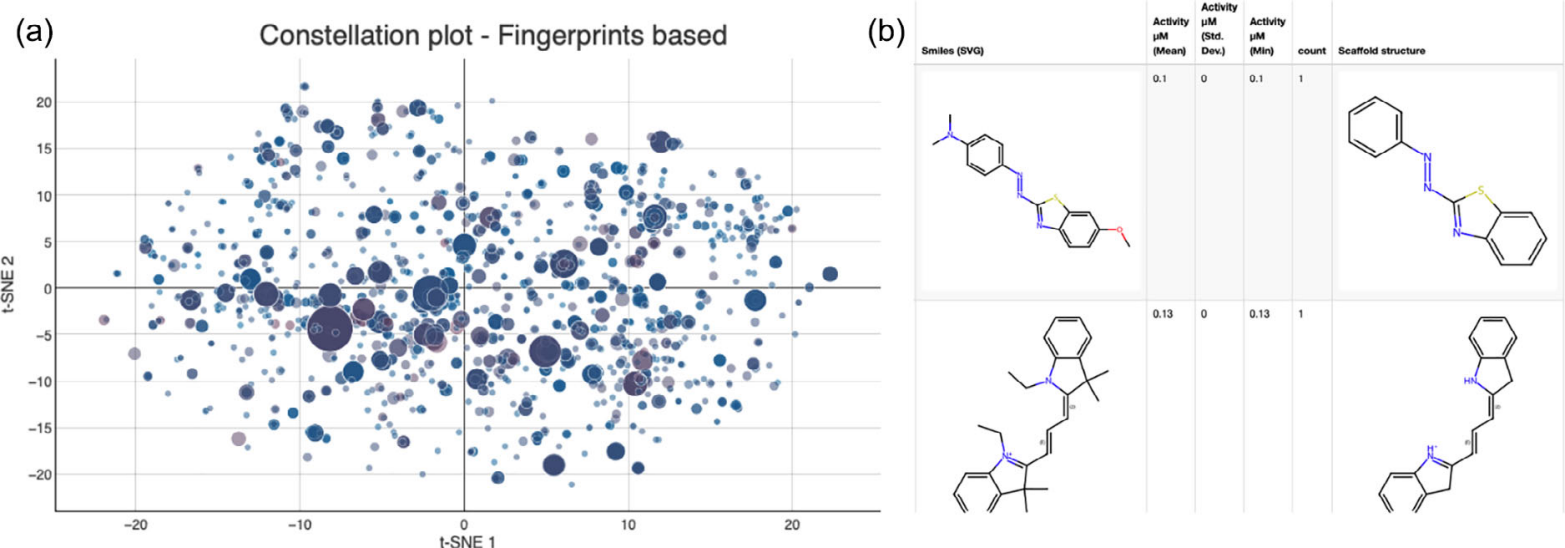
Interactive Scaffold‐based Constellation Plot. (a) Interactive visualization of the chemical space, each data point represents distinct scaffolds, the diameter of each data point indicates the scaffold frequency. The continuous color scale maps the ’Activity μM (Mean)’ (Blue: low activity; Red: high activity). (b) Dynamic datasheet viewer showing the leading rows and columns of the analyzed Tau‐DS.

The color‐coding component enables the user to color the data points by a specific numeric column. For the Tau‐DS Constellation Plot, the “Activity μM (Mean)” column was selected with a range of color from blue (min value) to red (max value), as illustrated in Figure 2.

The *Bubble Chart* (*Plotly*) node is configured with a ‘*Maximum number of rows*’ limit (predefined at 2000 to prevent overplotting); the *X*‐Axis and *Y*‐Axis are mapped to “*Coord_X*” and “*Coord_Y*”, respectively, with labels that automatically update according to the selected dimensionality reduction method (e.g., ‘t‐SNE 1′ for the *X*‐axis). Additionally, the ’Relative Size Column’ is assigned to the “count” column, allowing each data point to visually represent the frequency of scaffold occurrences.

For the *Table View* (*JavaScript*), the selection of the following columns is predefined for a correct information retrieval: *“Original Structure”*, *“Smiles* (*SVG*)*”*, “Activity μM (Rounded)”, “Activity μM (Mean)”, “Activity μM (Std. Dev.)”, “Activity μM (Min*)”, “count “, and “Scaffold Structure”.

A component was created with the nodes: *Bubble Chart* (*Plotly*) and *Table View* (*JavaScript*). See Figure S2 (Supporting Information).

To enhance visualization, the component's view layout consisted of a single row with two columns. The *Bubble chart* (*Plotly*) node was put on the left side, while the *Table View* (*JavaScript*) was put on the right side. Both nodes’ views were configured to “Aspect radio ‐ 4:3”.

Figure 2 illustrates the interactive scaffold‐based Constellation plot for the Tau‐DS.

The End‐user workflow interface (Figure 1b) streamlines the execution process, providing a structured sequence of steps to facilitate the complete workflow run.

## 4 Results

### 4.1 Interactivity

The Constellation Plots provide bidirectional interactivity. Selecting scaffolds on the plot displays the corresponding information in the interactive datasheet, while selections made in the datasheet are highlighted on the plot.

#### 4.1.1 Plot‐Based Interactivity

If the user wants to get some information based on the selection of a specific data point on the plot, first it is required to disable the ’Show only selected’ option on the hamburger icon (top right corner of the plot), but enable it on the “datasheet” (Figure S3 in the Supporting Information).

As an example of this plot‐based interactivity, by selecting and using the ‘*lasso selec*t’ tool (on the left upper side of the Constellation Plot) to select the biggest data point, the plot will automatically change the color intensity for all the data points except the chosen one. The “interactive datasheet” will also update with the corresponding scaffold information (Figure S4 in the Supporting Information).

The biggest data point (i.e., the most frequent scaffold) in the Tau‐DS is the benzene scaffold (49 occurrences), with a mean activity of 12.35 μM, standard deviation of 7.17 μM, and min activity of 1.26 μM. The specific example of a compound with this scaffold is CHEMBL288114, with an activity of 1.26 μM. Please note that when the user follows the plot‐based interactivity, the interactive datasheet shows all the compounds that share the same scaffold. In this case, the user will expect to see 49 different compounds sharing the benzene scaffold.

#### 4.1.2 Datasheet‐Based Interactivity

In this specific interactivity mode, the ’*show selected only*’ setting should be inverted: enabled for the plot and disabled for the datasheet. This configuration ensures the datasheet displays the entire library, while selecting a compound causes the plot to dynamically isolate its corresponding scaffold position.

For Tau‐DS, if the user selects a compound with the azobenzene scaffold in the datasheet, the plot automatically shows this scaffold position. In Tau‐DS, the azobenzene scaffold appears 29 times (the second most frequent scaffold), with a mean activity of 8.56 μM, standard deviation of 6.57 μM, and min activity of 0.56 μM. The specific example of a compound with this scaffold is CHEMBL1360012 with an activity of 7.94 μM (Figure S5 in the Supporting Information).

The Tau‐DS contains 5,211 compounds which share 3293 unique scaffolds (this unique scaffold number was retrieved from the *value counter* node in branch “(b)”).

### 4.2 Variations of the Constellation Plots

Section 2.1 describes the use of the *color‐coding* component to select the color scale for the plot (by “Activity μM (Mean)”, but by selecting other properties, the user can unlock Constellation Plot variations (Figure S6 in the Supporting Information), such as:

a. The Constellation Plot features gradient mapping based on the “Activity μM (Min),**”** enabling the user to identify the most potent compound in a group sharing the same scaffold. Within the Tau‐DS, the compound CHEMBL3193923 exhibited the highest potency (0.45 μM) among the 14 compounds sharing the “benzaldehyde phenylhydrazone” scaffold. Notably, this scaffold group had a standard deviation of 5.54 μM, suggesting a significant spread in activity despite the shared structural core.
b. Constellation Plot features gradient mapping based on the “Activity μM (Std. Dev.)”, enabling the user to identify scaffolds with high SAR (structure–activity relationship) variability, highlighting structural cores where chemical substitutions lead to significant fluctuations in biological potency. In Tau‐DS, the two compounds share the 1,3‐benzodioxole scaffold, CHEMBL1312986 (3.55 μM) and CHEMBL1331200 (31.62 μM). Their mean activity is 17.59 μM, but the standard deviation is 19.85 μM, which may reflect the importance of keeping rigid linkers instead of flexible ones, which can decrease activity by almost ten times.

The information obtained from the Constellation Plot not only helps the user to identify chemical modifications that might enhance potency but also encourages the exploration and optimization of understudied scaffolds, such as the 1,3‐benzodioxole core in Tau‐DS.

## 5 Conclusions

We present an automated, scaffold‐based Constellation Plot workflow implemented in the open‐source KNIME platform to generate interactive and reproducible visualizations of high‐dimensional chemical space. The workflow is designed for ease of use, helping bridge the gap between advanced chemoinformatics methods and their practical application by researchers with limited computational expertise. The utility of the workflow was demonstrated using a dataset of Tau protein inhibitors annotated with experimental activity, illustrating how the approach can support the exploration of SAR in scaffold‐based representation of chemical spaces. The workflow is openly available on GitHub, facilitating accessibility, reproducibility, and further integration into chemoinformatics studies.

## Supporting Information

Additional supporting information can be found online in the Supporting Information section.

## Conflicts of Interest

The authors declare no conflicts of interest.

## Supporting information

Supplementary Material

## Data Availability

The nodes used to build the presented workflow are open‐access and available for free use on the KNIME platform. Additionally, the workflow and Tau‐DS chemical database are available at https://github.com/Daniphantom99/KNIME_Constellation_plots.
