# Supplementary material for: Constellation Plots in KNIME: An Automated Scaffold‐Based Workflow for Interactive Chemical Space Visualization
Source: Mol Inform. 2026 Apr 30;45(5):e70035. doi: 10.1002/minf.70035 (PMC13129643; doi:10.1002/minf.70035)
Supplement: Supplementary file 1 — Supplementary Material [file MINF-45-e70035-s001.pdf]

Supporting Information

Constellation Plots in KNIME: An Automated Scaffold-Based Workflow for Interactive Chemical Space Visualization

Carlos D. Ramírez-Márquez[1], Edgar López-López[1,2] and José L. Medina-Franco[1]

[1] DIFACQUIM Research Group, Department of Pharmacy, School of Chemistry, Universidad Nacional Autónoma de México, Mexico City 04510, Mexico.

[2] Department of Pharmaceutical Biosciences, Uppsala University, Box 591, 75124, Uppsala, Sweden.

Note 1. Node warnings:

The RDKit Find Murcko Scaffolds node identifies acyclic compounds as null values (Warning: 'Found 'null' in a result cell - Replaced it with a missing cell'). This data gap propagates to the RDKit Fingerprint node, which triggers a warning ('Encountered empty input cell') due to the lack of structural input. Consequently, the t-SNE (L. Jonsson) node excludes these entries from the calculation ('1 row was ignored because it contained missing values').

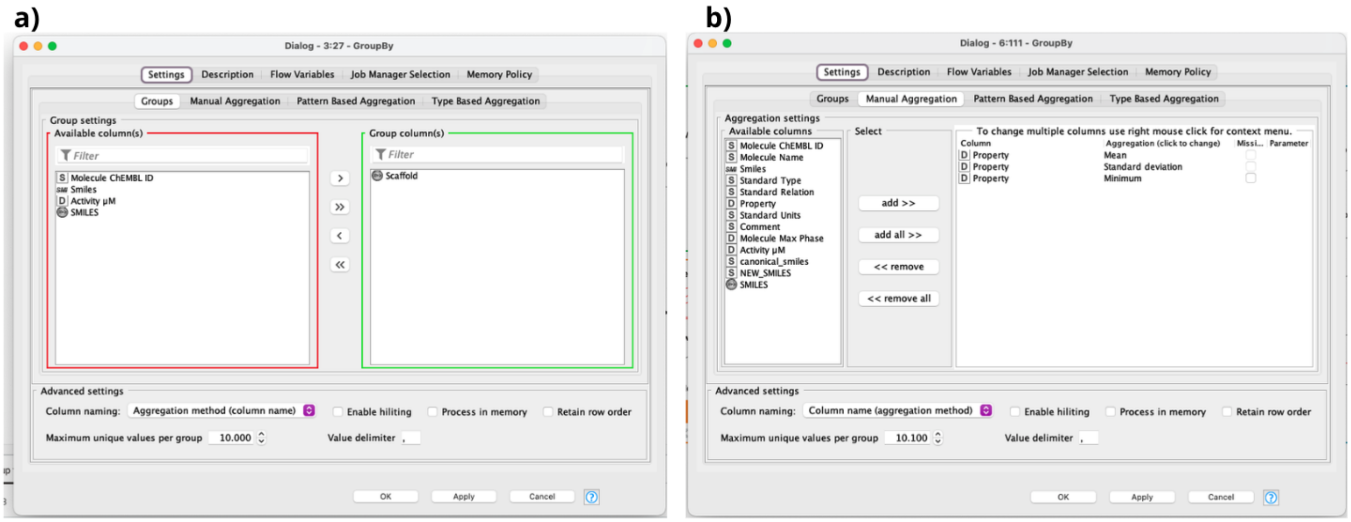

Figure S1. Configuration of the *GroupBy node*. a) Groups tab: Facilitates the selection of categorical or numerical variables from the *Available columns* to the *Group column(s)* section to define the basis of the aggregation. b) Manual Aggregation tab: Enables the definition of specific aggregation methods (e.g., Mean, Standard deviation, Minimum, and Maximum) for the selected attributes. The desired statistical measure can be selected by clicking on the *Aggregation* column header.

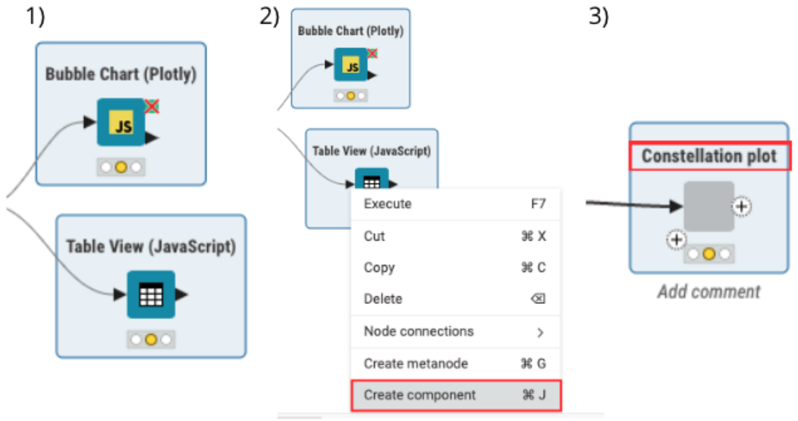

Figure S2. Detailed overview of the steps to create the KNIME component. 1) selection of both the Bubble chart (Ploty) node and the Table View (JavaScript) node, 2) right click - selection of "*Create component*" option, and 3) assign the name of the new component. The component is used to generate an interactive Constellation Plot.

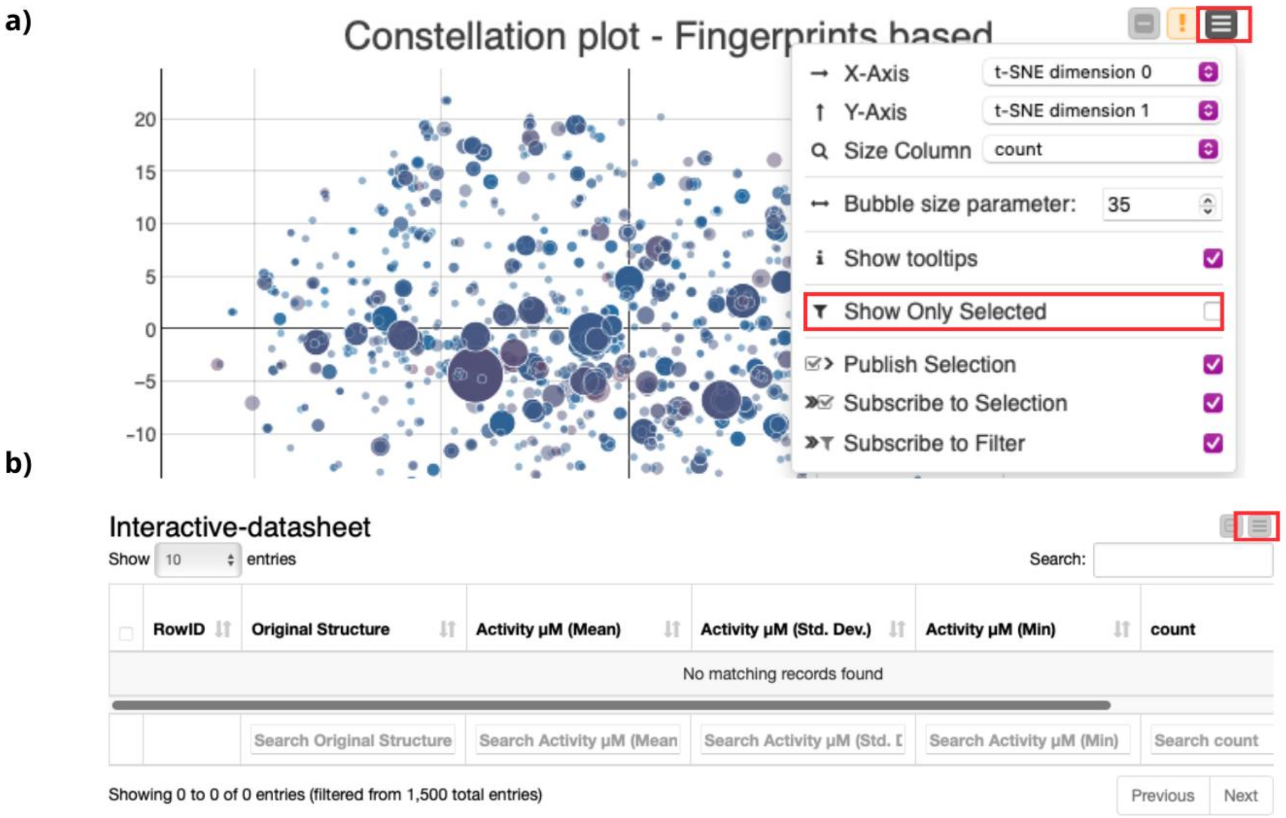

Figure S3. Detailed overview of the 'show only selection' option. By selecting the hamburger button, the option is shown on either the plot or the interactive datasheet. S3-a) On the plot's option, the user should disable the 'show only selection' option. S3-b) On the datasheet's option, the user should enable the 'show only selection' option.

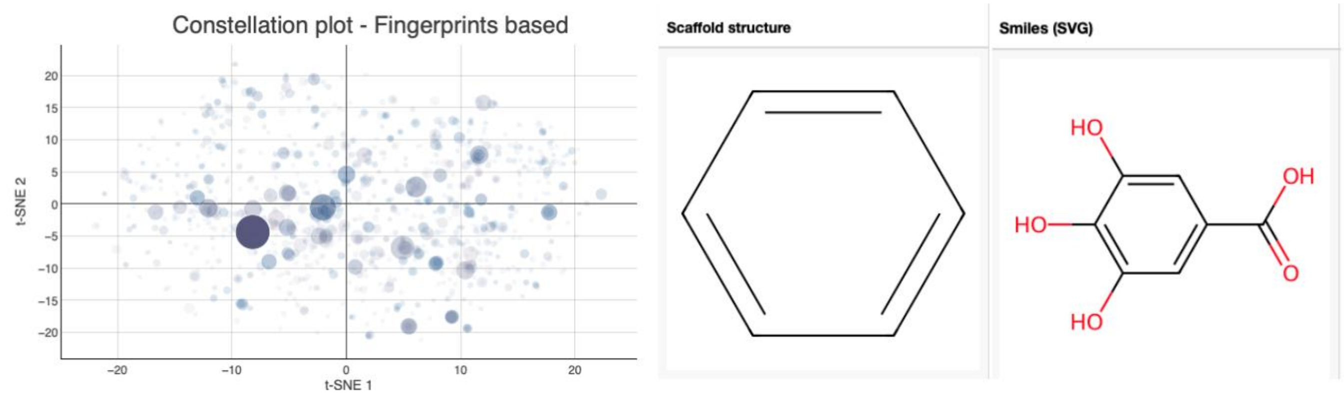

Original Structure	Activity µM	Activity µM (Rounded)	Mean(Activity µM)
CHEMBL288114	1.26	1.26	12.35

Standard deviation(Activity µM)	Min*(Activity µM)	PCA dimension 0	PCA dimension 1	count
7.17	1.26	-0.7517090668919116	0.38127043852905557	46

Figure. S4 Detailed overview of the plot-based interactivity. By selecting the largest data point, the interactive datasheet updates automatically, displaying information about the 46 compounds that share the benzene scaffold. The benzene has a mean activity of 12.35 µM, standard deviation of 7.17 µM, and a minimum activity of 1.26 µM. The specific example of a compound with this scaffold is CHEMBL288114, with an activity of 1.26 µM. The visual representation of this figure was optimized for enhanced clarity; no data was altered.

a)

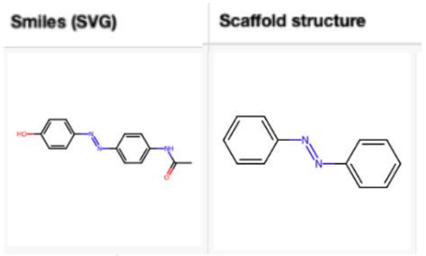

Original Structure	Activity (µM)	Mean(Activity (µM))
CHEMBL1360012	7.94	8.56

Standard deviation(Activity (µM))	Min*(Activity (µM))	count
6.57	0.56	29

b)

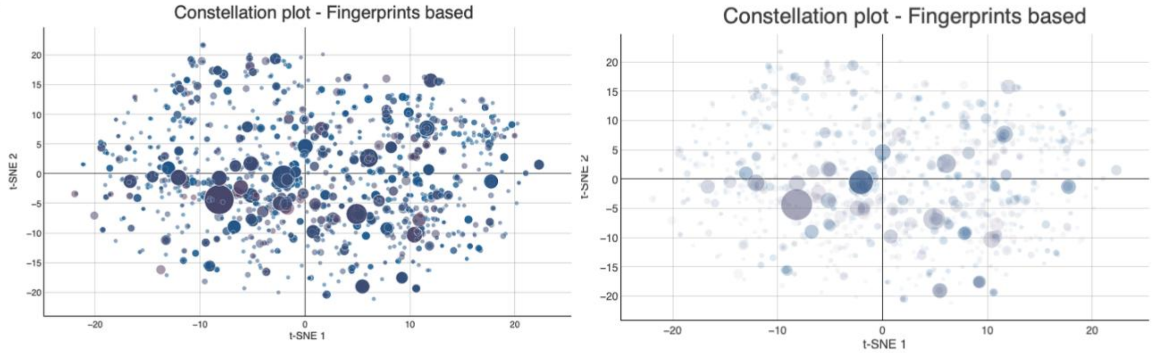

Figure S5. Detailed overview of the data sheet-based interactivity. (a) By selecting the "azobenzene" scaffold in the data sheet, the plot (b) updates automatically, showing the "azobenzene" scaffold position. The visual representation of this figure was optimized for enhanced clarity; no data was altered.

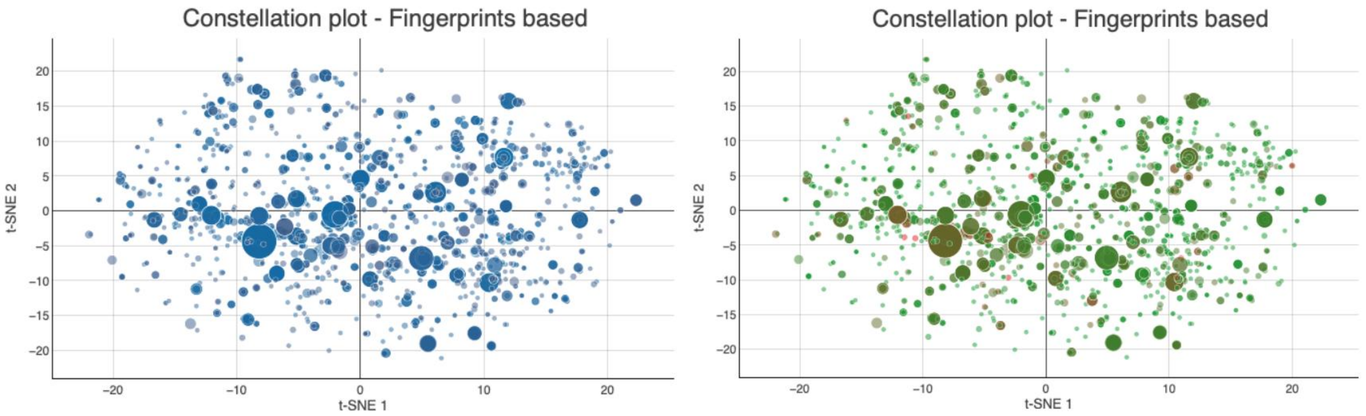

Figure S6. Variations of the Constellation Plots. *Left*: The Constellation Plot features gradient mapping based on the “Activity µM (Min)”- color gradient: blue: low activity; red: high activity. *Right*: Constellation Plot features gradient mapping based on the “Activity µM (Std. Dev.)” - color gradient: green: low deviation standard; red: high standard deviation.
